# Herb-Partitioned Moxibustion Regulates the TLR2/NF-*κ*B Signaling Pathway in a Rat Model of Ulcerative Colitis

**DOI:** 10.1155/2015/949065

**Published:** 2015-08-03

**Authors:** Xiaomei Wang, Yanan Liu, Hongsheng Dong, Luyi Wu, Xiaoming Feng, Zhigang Zhou, Chen Zhao, Huirong Liu, Huangan Wu

**Affiliations:** ^1^Yueyang Hospital of Integrated Traditional Chinese and Western Medicine, Shanghai University of Traditional Chinese Medicine, Shanghai 200437, China; ^2^Key Laboratory of Acupuncture-Moxibustion and Immunological Effects, Shanghai University of Traditional Chinese Medicine, Shanghai 200030, China

## Abstract

The TLR2/NF-*κ*B signaling pathway plays an important role in the pathomechanism of ulcerative colitis (UC); acupuncture and moxibustion can improve the damage in colonic tissues of UC, but the regulatory mechanism remains unknown. This study observed the effect of moxibustion on the TLR2/NF-*κ*B signaling pathway at the Tianshu (ST25) and Qihai (CV6) acupuncture points in the UC rat. The result shows that TLR2, IRAK1, and IKK-b mRNA and protein levels in the colonic mucosa were significantly higher in the UC rats than in the control rats. Herb-partitioned moxibustion reduced the expression of TLR2, IRAK1, and IKK-b mRNA and proteins in the UC rats. Similarly, the expression of NF-*κ*B was significantly increased and IFN-*β* and IL-10 were significantly decreased in the colonic mucosa of UC rats, but herb-partitioned moxibustion reduced the expression of IFN-*β* and upregulating the expression of IFN-*β* and IL-10 significantly. It indicates that herb-partitioned moxibustion can inhibit the expression of multiple signaling molecules of the TLR2 pathway effectively, and it may modulate the excessive local immune response by inhibiting TLR2 signaling, thereby promoting the repair of damaged colonic mucosa.

## 1. Introduction

Ulcerative colitis (UC), also known as nonspecific ulcerative colitis, is an inflammatory bowel disease (IBD) of unknown etiology. The incidence of IBD in China has shown a clear upward trend in recent years [1]. Although its pathogenesis is still largely unknown, progress in the study of the toll-like receptors (TLRs) and nuclear factor-*κ*B (NF-*κ*B) has improved understanding of the pathogenic process of UC. Under normal physiological conditions, intestinal epithelial cells can tolerate the commensal bacteria in the gut and maintain homeostasis. Studies have demonstrated that “tolerance” and “nontolerance” of the intestinal mucosa towards intestinal bacteria are dependent on TLR-mediated signaling pathways [2]. TLR pathways also play important roles in maintaining tissue integrity and repairing damaged tissues [2]. TLRs play an immunoregulatory role in the colonic mucosa, since they activate downstream signaling pathways upon recognition of pathogen-associated molecular patterns (PAMPs) on pathogenic microbes [3–7]. TLRs are pattern recognition receptors that recognize common antigens on pathogenic microorganisms and they play key roles in the innate immune response [8]. Growing knowledge of the TLR/NF-*κ*B pathway provides a new opportunity in the research of UC pathogenesis. Inhibiting key molecules of this pathway to block excessive inflammation may be a new direction in the treatment of UC [9].

Many clinical and experimental studies have clearly demonstrated that acupuncture has a protective effect on the intestinal mucosa [10, 11]. Long-term clinical and experimental studies have shown that herb-partitioned moxibustion is effective in the treatment of UC. It has been reported that TLR2 mRNA and protein are either not expressed or expressed at very low levels in the normal colonic mucosa of healthy human subjects but are upregulated in the colonic mucosa of patients with UC [12, 13]. Furthermore, the expression of TLR2 increases with the severity of the disease, as indicated by clinical and endoscopic examinations [12, 13]. In addition, there is more NF-*κ*B DNA binding activity in the nuclei of cells of the colonic mucosa of patients with UC than in those of other individuals [14, 15]. This suggests that activation of the TLR/NF-*κ*B signaling pathways is closely associated with the development of UC. Based on previous studies, the activity of the TLR2/NF-*κ*B pathway was here analyzed in UC rat colons to determine the therapeutic mechanisms of herb-partitioned moxibustion in UC. Upstream molecules TLR2, IRAK1, IKK-*β*, and NF-*κ*B and downstream molecules IFN-*β* and IL-10 served as markers.

## 2. Materials and Methods

### 2.1. Animals

Forty male Sprague-Dawley rats (specific-pathogen-free), weighing 120 ± 20 g, were purchased from the Experimental Animal Center of the Fudan University School of Medicine. The animals were housed for seven days to allow them to adapt to the new environment before experimentation, and all animals were in good health when experimentation began.

### 2.2. Generation of the Ulcerative Colitis (UC) Model and Intervention

The 40 SD rats were randomly divided into control, UC, UC with herb-partitioned moxibustion treatment, and UC with salicylazosulfapyridine (SASP) treatment groups. Except for rats in the control group, UC was induced in all rats using an immunological method combined with local irritation [16]. Adjuvant mixture (containing protein antigens released from UC colon patients) was injected into the front footpad, hind footpad, dorsa, inguinal, and abdominal cavities on days 0, 10, 17, 24, and 31, respectively. On day 38, rats were administered 3 mL of 3% formalin and a 2 mL enema of antigen fluid.

Rats in the control and UC groups did not receive any intervention, but they were sham-handled in the same way as rats in the intervention groups. Intervention was initiated immediately after UC generation. For herb-partitioned moxibustion, premade medicinal cakes (diameter 0.5 cm, height 0.3 cm) were placed on the Tianshu (ST25) and Qihai (CV6) acupuncture points [17], and moxa cones (Nanyang Hanye Moxa Plant, Henan, China) about 90 mg in size were placed on the medicinal cakes. The moxa cones were ignited and allowed to burn out. Two cones were used for each acupuncture point once daily for a total of 14 times. For SASP treatment, salicylazosulfapyridine (SASP) (Lot# 201007C30, Sunve Pharmaceutical Ltd., Shanghai, China) solution was administered by gavage. The dose was determined with the ratio of an adult human (70 kg body weight) per rat (200 g body weight) at 1 : 0.018 (*Pharmacological Experimental Methodology*). The SASP solution was administered twice daily, 3 mL each time, for a total of 14 days.

### 2.3. Tissue Collection

At the end of the intervention, animals were killed by cervical dislocation and their abdomens were opened. The colon was quickly removed (about 8 cm from the anus toward the proximal end) and dissected free of the surrounding connective tissues and fat. It was then cut open lengthwise, washed in saline, and laid flat in a dish mucosa side up, and the gross morphology of the mucosa was examined by eye. The colon was then divided in half. One piece was fixed in 10% neutral-buffered formalin, paraffin-embedded, and cut into sections. The other piece was immediately frozen in liquid nitrogen and stored at −80°C for RNA preparation and qRT-PCR analysis.

### 2.4. UC Disease Monitor and Marker Analysis

#### 2.4.1. Monitoring of General Health Conditions of Rats

Rats were monitored for their demeanor, food and water intake, body weight, appearance of fur, responsiveness, activity, appearance of feces, and other body conditions.

#### 2.4.2. Morphological and Histopathological Examination of Rat Colon Mucosa

The colon tissue from the anus to the ileocecal region was dissected and its gross morphology was examined by eye. Tissue sections were stained by H + E and observed under an optical microscope to further examine the morphology of rat colonic mucosa.

#### 2.4.3. Fluorescence Quantitative RT-PCR (qRT-PCR) Analysis

(1) RNA extraction: rat colon tissues were removed from liquid nitrogen, and 1 mL TRIzol Reagent was added to every 100 mg of tissue. The tissue was homogenized twice for 15 s each, with a 10 s interval. 0.2 mL chloroform was added per 1 mL TRIzol Reagent, and then this mixture was vigorously shaken for 15 s, cooled on ice for 5 min, and centrifuged at 12,000 rpm and 4°C for 15 min in a microcentrifuge (Thermo, US). The upper aqueous phase was taken into another microcentrifuge tube, and an equal volume of prechilled isopropanol was added. The two solutions were slowly mixed, cooled on ice for 15–20 min, and then centrifuged at 12,000 rpm, 4°C for 10 min. The pellet was washed with 75% ethanol (DEPC-water : ethanol = 1 : 3) and centrifuged again at 7,500 rpm, 4°C for 8 min. The supernatant was removed, and the tubes were blotted dry on sterilized filter paper. The pellet was washed again, the ethanol was removed, and the pellet was air-dried. After the pellet was dry; it was dissolved in DEPC-water. Then 1 mL of total RNA solution was used to determine RNA concentration and purity using NanoDrop. (2) Primer design and synthesis: primer 5 software was used to design qRT-PCR primers for the genes TLR2, IRAK1, IKK-*β*, and *β*-actin (Table 1). Primers were synthesized and purified by Sangon Biotech (Shanghai, China). (3) cDNA synthesis by one-step reverse transcription: here, 16.0 *μ*L 5x iScript reaction mix, 4.0 *μ*L iScript reverse transcriptase, 4 *μ*g total RNA (volume depends on the concentration), and 60.0 *μ*L DEPC-H_2_O 60.0 *μ*L were added to each 0.5 mL RNase-free centrifuge tube to a total reaction volume of 80 *μ*L. The reaction program was as follows: 25°C for 5 min, 42°C for 30 min, and 85°C for 5 min. (4) qPCR reaction: here, 1.0 *μ*L iQ SYBR Green Supermix, 1.0 *μ*L cDNA, 1.0 *μ*L each sense and antisense primer, and 8 *μ*L ddH_2_O were added to each well of 0.2 mL 96-well PCR plates. The plates were centrifuged briefly and placed into a PCR machine (LC96 PCR system, Roche, Switzerland) for amplification. The PCR program was (1) 95°C for 5 min and (2) 40 cycles of 95°C for 10 s, 58°C for 30 s, and 72°C for 10 s.

#### 2.4.4. Immunohistochemical Detection of TLR2, IRAK1, IKK-*β*, NF-*κ*B, IFN-*β*, and IL-10 Proteins in Colonic Mucosa

Tissue sections were deparaffinized with the following solutions: three changes of xylene, each lasting for 10 min: 100%, 95%, and 85%; 75% ethanol for 2 min and then placing in water. Sections were washed three times with 0.01 M PBS (pH 7.4) for 3 min each and then incubated with 1% H_2_O_2_ for 20 min. Then they were washed three times in PBS for 3 min each and three distilled water washes, 3 min each. Sections were incubated with PBS for 5 min and then subjected to antigen retrieval. For antigen retrieval, the sections were placed in 0.01 M citrate buffer (pH 6.0) and boiled three times in a microwave at setting III (98°C) for 2.5 min, 1.5 min, and 1 min, respectively. Sections were kept in the hot buffer for 15 min each between rounds of boiling. Sections were let to cool at room temperature and then washed twice with distilled water, followed by three washes with PBS, 3 min each. Sections were blocked in 5% goat serum at room temperature for 20 min and then incubated with diluted primary antibodies (30–50 *μ*L) at 4°C overnight. The next day, sections were washed three times with PBS for 3 min each and then incubated with biotinylated secondary antibody-EnVision reagent (30–50 *μ*L) for 30 min at 37°C, followed by three washes with PBS, 3 min each. To allow visualization of bound antibodies, sections were incubated with 0.04% DAB + 0.03% H_2_O_2_ for 8 min for color development. Sections were washed in water, counterstained with hematoxylin for 30 s, washed again with water, dipped in a bluing solution containing hydrochloric acid and ethanol for 2 s, washed in water, and mounted with resin.

Stained sections were examined under a light microscope. Positive staining was brown, and counterstained nuclei were blue. Three randomly chosen microscopic fields were analyzed with the MOTIC image analysis system. Total area and integrated optical density of positive signals were measured for each field, and the mean values from the three fields of each sample were used for statistical analysis.

### 2.5. Statistical Analysis

Quantitative data are here presented as mean ± standard deviation ($\overset{-}{x}\pm s$), and statistical analysis was performed with the PASS 13.0 software. Comparisons between groups were conducted using one-way ANOVA with the following methods: LSD/SNK-q method was used when pairwise tests showed that the variances of different groups were equal, but Dunnett's T3 method was used when the variances were not equal. The level of significance was set to *α* = 0.05 and *P* < 0.05.

## 3. Results

### 3.1. General Health Conditions of Rats in the Experimental Groups

Rats in the control group had normal food and water intake, were active, and had dense and shiny pelts. UC rats showed debility and anorexia, their food intake was reduced, and they had a hunched posture. They showed reduced activity levels and were easily startled. Their feces appeared normal and their perianal skin remained clean. Their coats appeared rough and less shiny. They had increased stool frequency, bloody mucus was seen in the feces, and the perianal skin was dirty with feces. The toes injected with antigen were bruised and swollen. One rat in this group died during the experimental period. Rats in the herb-partitioned moxibustion and SASP treatment groups displayed better food intake, responsiveness, activity levels, and appearance of the fur coat than rats in the UC group.

**Figure 1:**
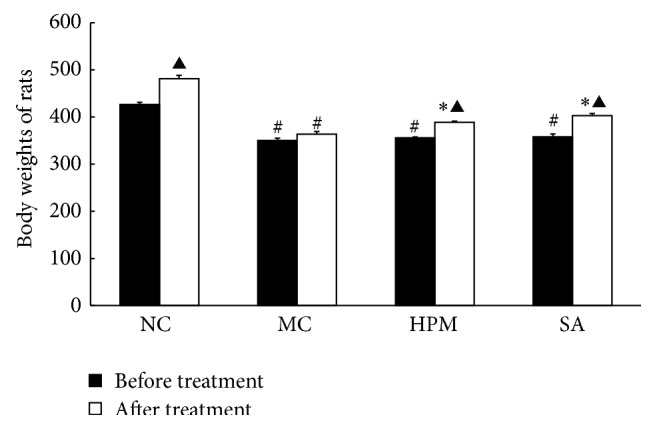
The body weights of rats in each group. NC: normal control; MC: ulcerative colitis; HPM: UC with herb-partitioned moxibustion; SA: UC with salicylazosulfapyridine. ^#^ *P* < 0.01 versus NC; ^*∗*^ *P* < 0.01 versus MC; ^▲^ *P* < 0.01 versus before treatment.

As shown in Figure 1, rats in the control group gained significantly weight by the end of the experimental period (*P* < 0.01). The body weights of UC rats were significantly lower than those of the control rats (*P* < 0.01). The body weights of rats in the herb-partitioned moxibustion and SASP treatment groups were greater than those of rats in the UC group (*P* < 0.01).

### 3.2. Macro- and Microscopic Morphology of Rat Colonic Mucosa

Observed by the naked eye, the colonic mucosa of control rats had a smooth surface with small amounts of mucus. The blood vessels underneath the mucosal folds were visible, and there was no erosion or ulceration. The colonic mucosa of UC rats had severe hyperemia, edema, and erosion, and ulcers were observed. In contrast, the appearance of colonic mucosa from rats in the herb-partitioned moxibustion and SASP treatment groups was markedly better than those of other groups. Even though the mucosal surface was not as smooth as in normal mucosa and there was some visible edema, the muscles under the mucosal folds were visible and the extent of hyperemia, edema, and erosion of the mucosa was significantly less pronounced than in UC rats.

**Figure 2:**
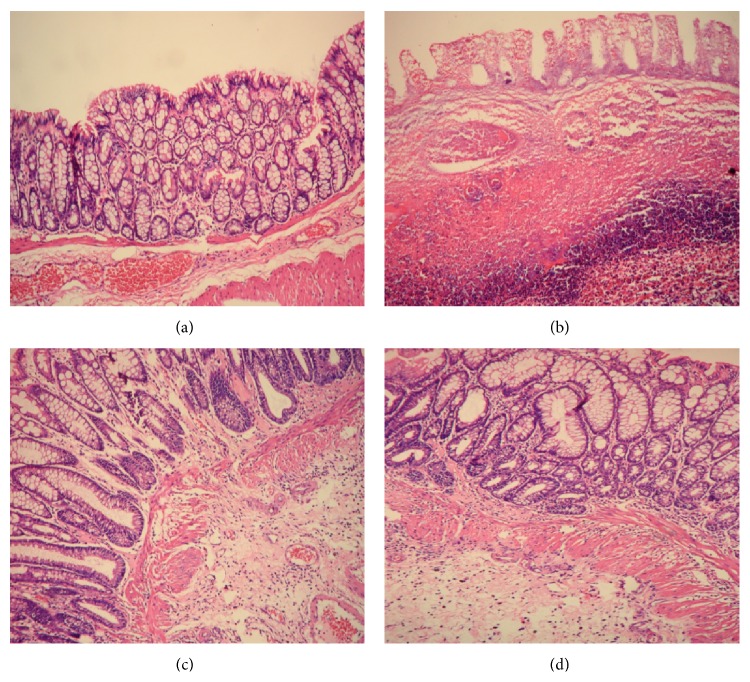
The histological observation of rats in each group. Hematoxylin-Eosin staining method, ×200. (a) Normal control; (b) ulcerative colitis; (c) UC with herb-partitioned moxibustion; (d) UC with salicylazosulfapyridine.

As shown in Figure 2, under a light microscope, normal colonic mucosa had a well-organized structure with intestinal glands arranged in rows and an intact colonic epithelium. Capillaries and scattered lymphocytes were visible in the lamina propria, but there was no significant inflammatory cell infiltration. In the colonic mucosa of UC rats, the crypts were shorter and the epithelium was not intact. The mucosa and submucosa had been infiltrated by a large number of inflammatory cells, focal hyperemia and edema were visible, and the structures of the glands were disorganized, indicating that ulcers had formed. Both herb-partitioned moxibustion and SASP treatments greatly improved the morphology of colonic mucosa. Intestinal glands were more organized than those in UC rats. Ulceration was less severe, and the mucosa surface was covered by epithelial cells. Edema and inflammatory cell infiltration in submucosa were still present, but they were less severe than in UC rats.

### 3.3. TLR2 mRNA and Protein Expression in Rat Colonic Mucosa

**Figure 3:**
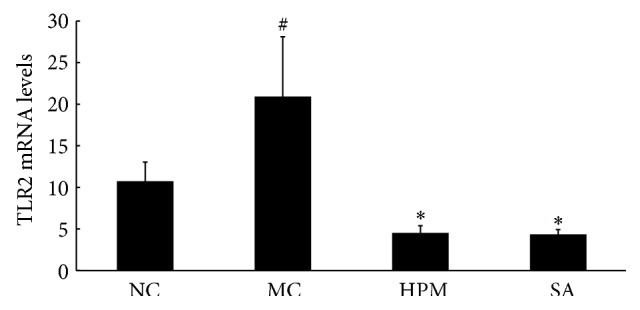
The TLR2 mRNA levels in the rat colon of each group. NC: normal control; MC: ulcerative colitis; HPM: UC with herb-partitioned moxibustion; SA: UC with salicylazosulfapyridine. ^#^ *P* < 0.01 versus NC; ^*∗*^ *P* < 0.01 versus MC.

As shown in Figure 3, TLR2 mRNA level in the colonic mucosa was significantly higher in the UC rats than in the control rats (*P* < 0.01). Both herb-partitioned moxibustion and SASP treatments significantly reduced TLR2 mRNA expression in the colonic mucosa of UC rats (*P* < 0.01). This inhibitory effect was slightly greater with SASP treatment than with herb-partitioned moxibustion, but the difference was not statistically significant (*P* > 0.05).

**Figure 4:**
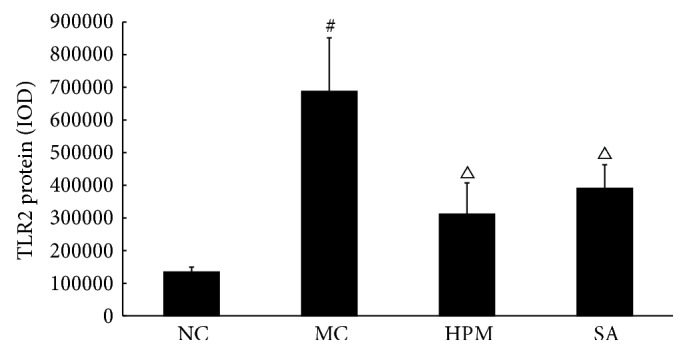
Expression of TLR2 protein in the colonic mucosa of rat. NC: normal control; MC: ulcerative colitis; HPM: UC with herb-partitioned moxibustion; SA: UC with salicylazosulfapyridine. ^#^ *P* < 0.01 versus NC; ^△^ *P* < 0.05 versus MC.

**Figure 5:**
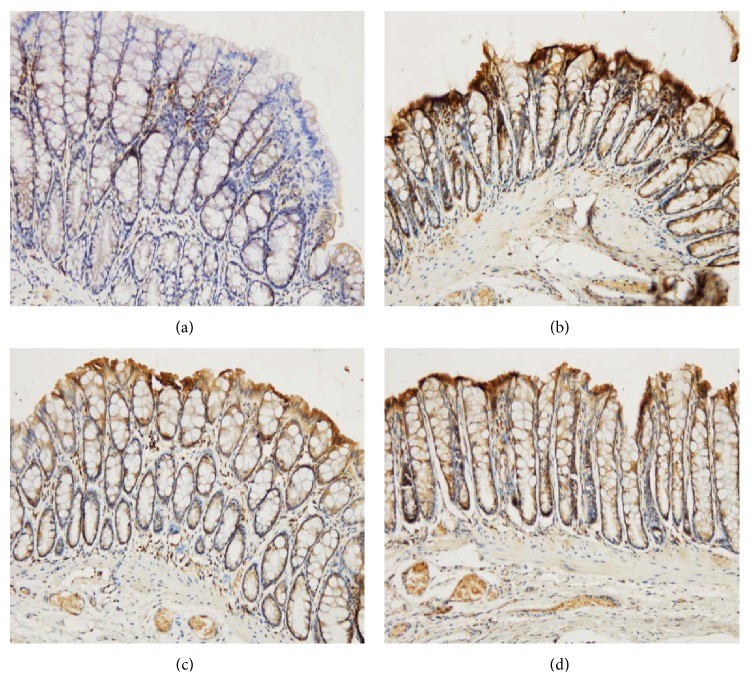
The integral optical density (IOD) of TLR2 in each rat group. EnVision Plus method, ×200. (a) Normal control; (b) ulcerative colitis; (c) UC with herb-partitioned moxibustion; (d) UC with salicylazosulfapyridine.

TLR2 protein expression was determined using immunohistochemistry and quantified by the total area of staining and the integrated optical density. As shown in Figures 4 and 5, TLR2 protein levels in the colonic mucosa were higher in UC rats than in control rats, as indicated by both of these measurements (*P* < 0.01). The total area of TLR2 staining was significantly lower in both the herb-partitioned moxibustion and SASP treated groups than in the UC group (*P* < 0.01, *P* < 0.05). The integrated optical density was also significantly lower in both treatment groups (*P* < 0.05). TLR2 protein levels were slightly lower in the herb-partitioned moxibustion group than in the SASP group, but the difference was not statistically significant (*P* > 0.05).

### 3.4. IRAK1 mRNA and Protein Expression in Rat Colonic Mucosa

**Figure 6:**
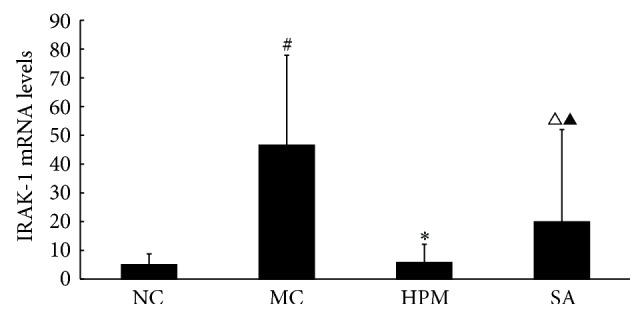
The IRAK-1 mRNA levels in the rat colon of each group. NC: normal control; MC: ulcerative colitis; HPM: UC with herb-partitioned moxibustion; SA: UC with salicylazosulfapyridine. ^#^ *P* < 0.01 versus NC; ^*∗*^ *P* < 0.01, ^△^ *P* < 0.05 versus MC; ^▲^ *P* < 0.01 versus HPM.

As shown in Figure 6, rats in the UC group expressed significantly higher levels of IRAK1 mRNA in the colonic mucosa than rats in the control group (*P* < 0.01). Both herb-partitioned moxibustion and SASP treatments significantly reduced IRAK-1 mRNA expression in the colonic mucosa of UC rats (*P* < 0.01, *P* < 0.05). Between the two treatment groups, IRAK1 mRNA expression in the colonic mucosa was significantly higher with SASP than with herb-partitioned moxibustion treatment (*P* < 0.01).

**Figure 7:**
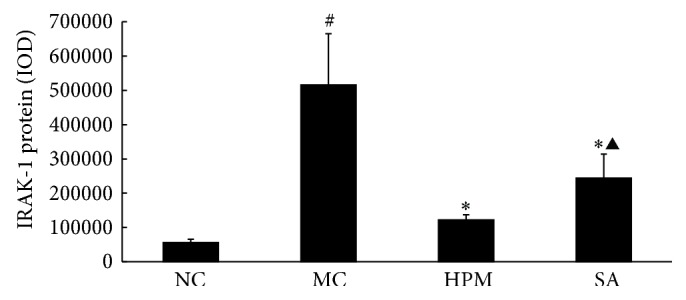
Expression of IRAK-1 protein in the colonic mucosa of rat. NC: normal control; MC: ulcerative colitis; HPM: UC with herb-partitioned moxibustion; SA: UC with salicylazosulfapyridine. ^#^ *P* < 0.01 versus NC; ^*∗*^ *P* < 0.01 versus MC; ^▲^ *P* < 0.01 versus HPM.

**Figure 8:**
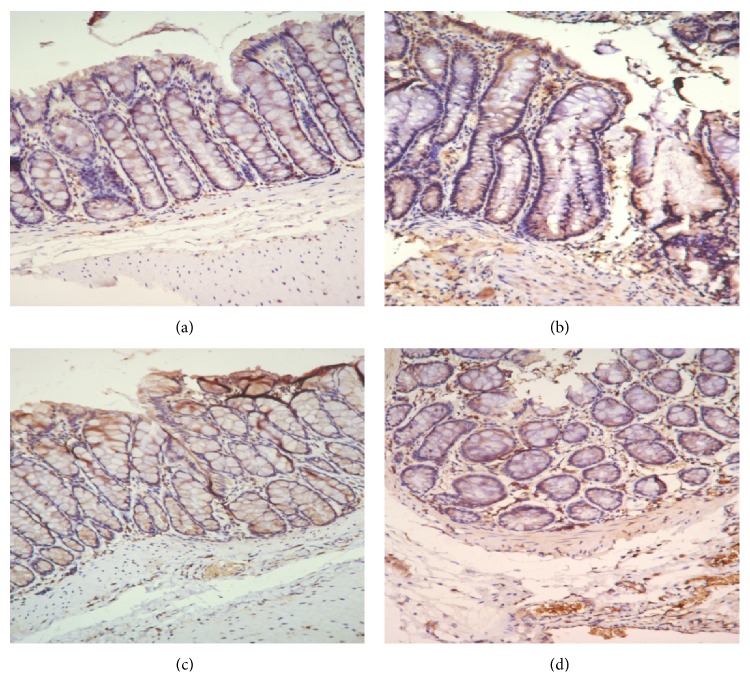
The integral optical density (IOD) of IRAK-1 in each rat group. EnVision Plus method, ×200. (a) Normal control; (b) ulcerative colitis; (c) UC with herb-partitioned moxibustion; (d) UC with salicylazosulfapyridine.

As shown in Figures 7 and 8, IRAK1 protein levels in the colonic mucosa were significantly higher in UC rats than in the control rats, as measured by both the total area of staining and the integrated optical density (*P* < 0.01 in both cases). Both herb-partitioned moxibustion and SASP treatments significantly reduced IRAK1 protein levels in the colonic mucosa of UC rats, as indicated by both of these measurements (*P* < 0.01 in all cases). IRAK1 protein levels in the colonic mucosa were significantly lower in the herb-partitioned moxibustion than in the SASP treatment by both of these measurements (*P* < 0.01 in both cases).

### 3.5. IKK-*β* mRNA and Protein Expression in Rat Colonic Mucosa

**Figure 9:**
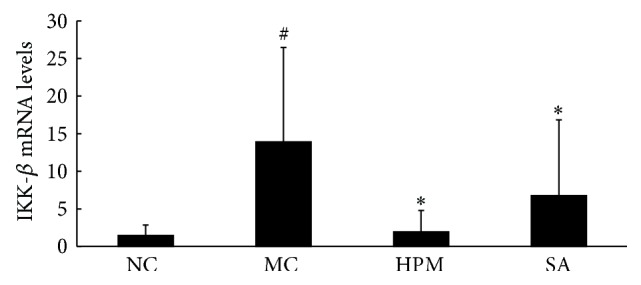
The IKK-*β* mRNA levels in the rat colon of each group. NC: normal control; MC: ulcerative colitis; HPM: UC with herb-partitioned moxibustion; SA: UC with salicylazosulfapyridine. ^#^ *P* < 0.01 versus NC; ^*∗*^ *P* < 0.01 versus MC.

As shown in Figure 9, rats in the UC group expressed significantly higher levels of IKK-*β* mRNA in the colonic mucosa than rats in the control group (*P* < 0.01). Both herb-partitioned moxibustion and SASP treatments significantly reduced IKK-*β* mRNA expression in the colonic mucosa of UC rats (*P* < 0.01).

**Figure 10:**
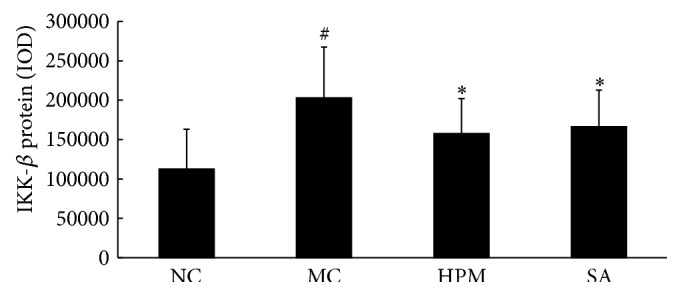
Expression of IKK-*β* protein in the colonic mucosa of rat. NC: normal control; MC: ulcerative colitis; HPM: UC with herb-partitioned moxibustion; SA: UC with salicylazosulfapyridine. ^#^ *P* < 0.01 versus NC; ^*∗*^ *P* < 0.01 versus MC.

**Figure 11:**
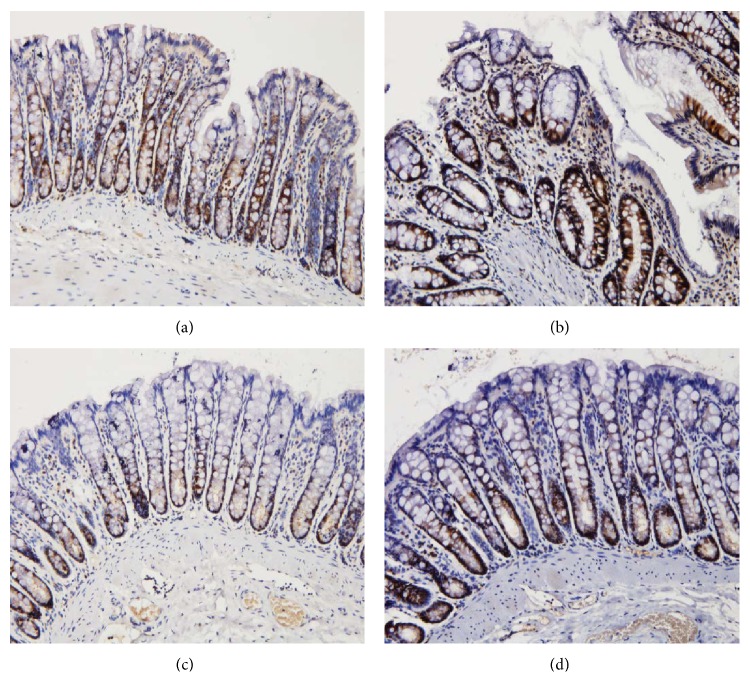
The integral optical density (IOD) of IKK-*β* in each rat group. EnVision Plus method, ×200. (a) Normal control; (b) ulcerative colitis; (c) UC with herb-partitioned moxibustion; (d) UC with salicylazosulfapyridine.

As shown in Figures 10 and 11, IKK-*β* protein levels in the colonic mucosa were significantly higher in UC rats than in the control rats, as measured by both the total area of staining and the integrated optical density (*P* < 0.05, *P* < 0.01). Both herb-partitioned moxibustion and SASP treatments significantly reduced IKK-*β* protein levels in the colonic mucosa of UC rats, as indicated by both of these measurements (*P* < 0.05).

### 3.6. NF-*κ*B Expression in Rat Colonic Mucosa

**Figure 12:**
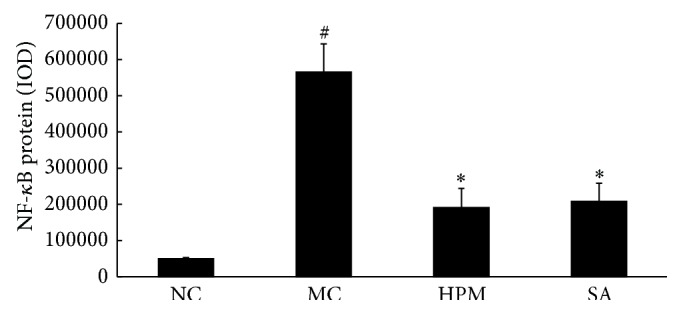
Expression of NF-*κ*B protein in the colonic mucosa of rat. NC: normal control; MC: ulcerative colitis; HPM: UC with herb-partitioned moxibustion; SA: UC with salicylazosulfapyridine. ^#^ *P* < 0.01 versus NC; ^*∗*^ *P* < 0.01 versus MC.

**Figure 13:**
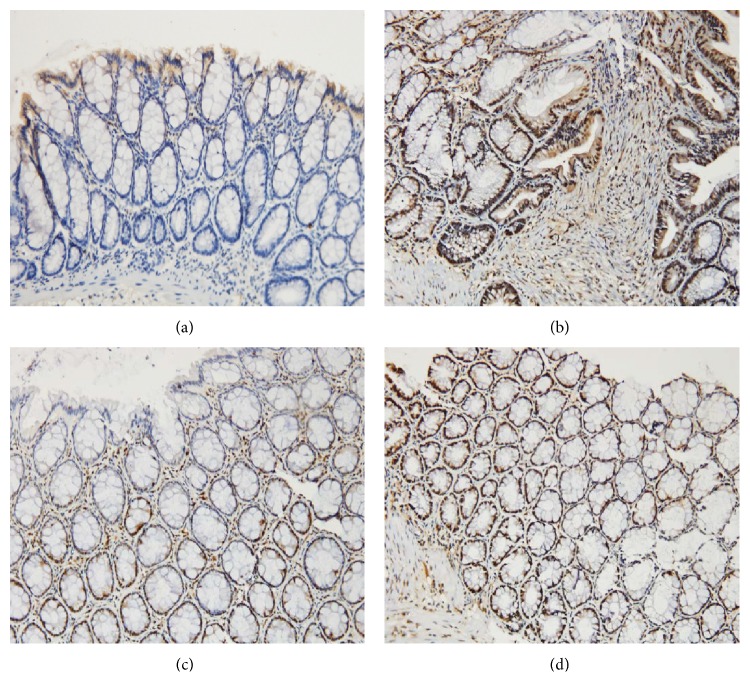
The integral optical density (IOD) of NF-*κ*B in each rat group. EnVision Plus method, ×200. (a) Normal control; (b) ulcerative colitis; (c) UC with herb-partitioned moxibustion; (d) UC with salicylazosulfapyridine.

As shown in Figures 12 and 13, NF-*κ*B protein levels in the colonic mucosa were significantly higher in UC rats than in the control rats, as measured by both the total area of staining and the integrated optical density (*P* < 0.01). Both herb-partitioned moxibustion and SASP treatments were associated with lower NF-*κ*B protein levels in the colonic mucosa of UC rats as indicated by the total area of staining (*P* < 0.01). NF-*κ*B protein levels were slightly lower in the herb-partitioned moxibustion group than in the SASP group, but the difference was not statistically significant (*P* > 0.05).

### 3.7. IFN-*β* Expression in Rat Colonic Mucosa

**Figure 14:**
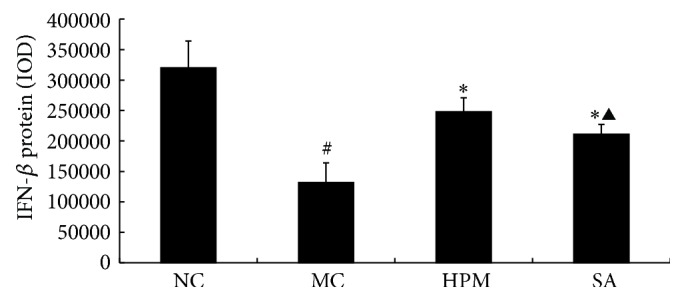
Expression of IFN-*β* protein in the colonic mucosa of rat. NC: normal control; MC: ulcerative colitis; HPM: UC with herb-partitioned moxibustion; SA: UC with salicylazosulfapyridine. ^#^ *P* < 0.01 versus NC; ^*∗*^ *P* < 0.01 versus MC; ^▲^ *P* < 0.01 versus HPM.

**Figure 15:**
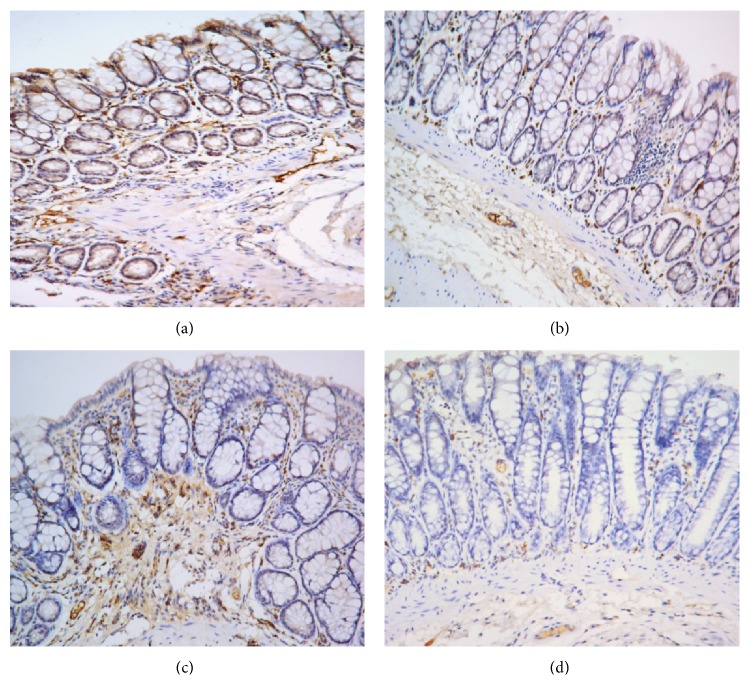
The integral optical density (IOD) of IFN-*β* in each rat group. EnVision Plus method, ×200. (a) Normal control; (b) ulcerative colitis; (c) UC with herb-partitioned moxibustion; (d) UC with salicylazosulfapyridine.

As shown in Figures 14 and 15, IFN-*β* protein levels in the colonic mucosa were significantly lower in UC rats than in the control rats, as indicated by both the total area of staining and the integrated optical density (*P* < 0.01 in both cases). Both herb-partitioned moxibustion and SASP treatments significantly increased IFN-*β* protein levels in the colonic mucosa of UC rats by both of these measurements (*P* < 0.01 in both cases). Between the two treatment groups, IFN-*β* protein levels in the colonic mucosa were significantly higher with herb-partitioned moxibustion than with SASP treatment as indicated by both of these measurements (*P* < 0.05 in both cases).

### 3.8. IL-10 Expression in Rat Colonic Mucosa

**Figure 16:**
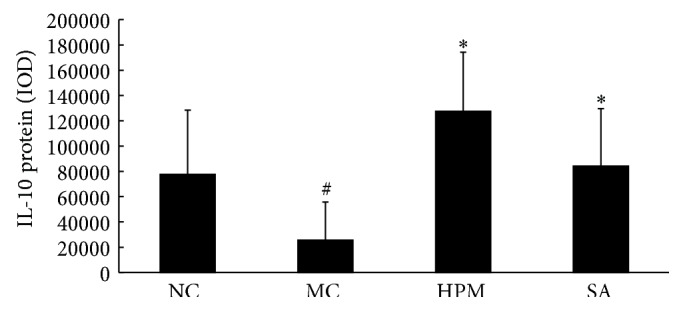
Expression of IL-10 protein in the colonic mucosa of rat. NC: normal control; MC: ulcerative colitis; HPM: UC with herb-partitioned moxibustion; SA: UC with salicylazosulfapyridine. ^#^ *P* < 0.01 versus NC; ^*∗*^ *P* < 0.01 versus MC.

**Figure 17:**
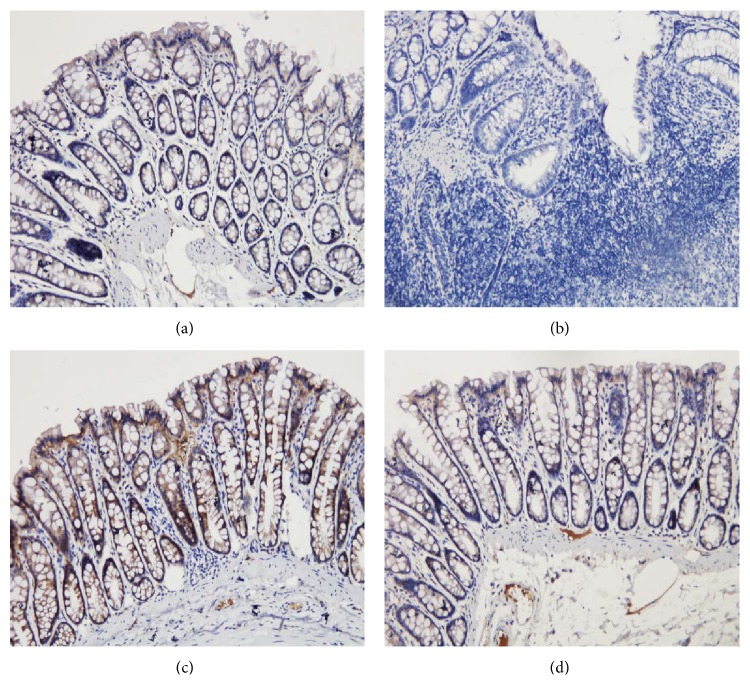
The integral optical density (IOD) of IL-10 in each rat group. EnVision Plus method, ×200. (a) Normal control; (b) ulcerative colitis; (c) UC with herb-partitioned moxibustion; (d) UC with salicylazosulfapyridine.

As shown in Figures 16 and 17, IL-10 protein levels in the colonic mucosa were significantly lower in UC rats than in the control rats, as indicated by both the total area of staining and the integrated optical density (*P* < 0.05 in both cases). Both herb-partitioned moxibustion and SASP treatments significantly increased IL-10 protein levels in the colonic mucosa of UC rats by both of these measurements (*P* < 0.05 in all cases). The total area of IL-10 staining in the colonic mucosa was significantly higher in the herb-partitioned moxibustion group than in the SASP group (*P* < 0.05) but the integrated optical density did not reach statistical significance, although it was higher in the herb-partitioned moxibustion group than in the SASP group (*P* > 0.05).

## 4. Discussion

According to its clinical manifestations in the traditional Chinese medicine, ulcerative colitis belongs to scope of “diarrhea” and “abdominal pain.” It occurs in the large intestine, but the basic pathogenesis is spleen deficient and hyperactivity of damp. Tianshu (ST25) acupoint, located at 2 inches next to navel of the stomach channel of foot-yangming, is the Mu point of large intestine, it is a key point regulating the ascending and descending Qi and is usually used to treat the abdominal pain and diarrhea [18], and it was recorded in ancient medical books, such as “Qianjin Yaofang.” Qihai (CV6) acupoint is located at 1.5 inches below navel of Ren meridian and the place gathering original Qi; it can regulate the ascending and descending Qi and complement original Qi of the human body. Therefore, moxibustion at ST25 and CV6 can improve the symptoms of diarrhea and abdominal pain in patient with ulcerative colitis, not only regulating Qi and reinforcing spleen and transporting dampness, but also complementing original Qi to promote recovery of patient.

Ulcerative colitis is associated with multiple aspects of immune dysfunction, including autoimmunity and dysregulated humoral and cellular immunity. It has been recognized that immune dysfunction plays an important role in the pathogenesis of UC [19–22].

The activation of TLR signaling pathways is involved in the development of UC. TLR2 activates NF-*κ*B through the MyD88-dependent pathway [23]. Activated NF-*κ*B induces the expression of TLR2 transcriptionally, resulting in a TLR2-NF-*κ*B-TLR2 positive feedback loop that propagates the inflammatory response [24]. The IRAK1 family protein acts as an adaptor protein and mediates TLR-induced production of proinflammatory cytokines. Degradation of IRAK1 comprises an important negative feedback mechanism that prevents excessive inflammation [25, 26]. The IKK*β*/NF-*κ*B signaling pathway plays key roles in inducing the immune response downstream of TLRs. IKK-*β* is an essential catalytic subunit of the protein complex that activates NF-*κ*B in response to inflammatory cytokines, such as TNF-*α*, IL-1, and lipopolysaccharide. Studies have shown that TLR2 expression is low in epithelial cells of normal intestinal mucosa but becomes high during inflammation [27]. Abnormally high expression of TLR2 was observed in the colonic mucosa of patients with UC and in mouse models of UC [28, 29]. In addition, the DNA binding activity of NF-*κ*B in cell nucleus was found to be higher in the colonic mucosa of patients with UC [30, 31]. These discoveries suggest that the activation of the TLR2/NF-*κ*B signaling pathway is closely associated with the development of UC.

Proinflammatory cytokines TNF-*α* and IL-12 and anti-inflammatory cytokines IL-10 and IFN-*β* play important roles in the pathogenesis of UC. In the presence of IL-6 and TNF-*α* causes microvascular dysfunction in the intestinal mucosa, damaging its barrier function. TNF-*α* also acts synergistically with IFN-*γ* to alter the structure and the barrier function of the intestinal mucosal epithelium, leading to increased permeability of the colonic mucosa and apoptosis of epithelial cells [32]. IL-12 stimulates the differentiation of T cells into IFN-*γ*-secreting T-helper type 1 (Th1) cells, which promotes inflammation by activating macrophages and neutrophils [24]. IL-10 is mainly produced by Th2 cells, activated B cells, monocytes, and macrophages. It dampens immune responses by antagonizing inflammatory mediators. IFN-*β* is a cytokine produced by fibroblasts and white blood cells. It can inhibit the proliferation of fibroblasts, epithelial cells, endothelial cells, and hematopoietic cells [33]. IFN-*β* plays an immunoregulatory role by promoting the secretion of IL-10 and T lymphocytes [34]. Studies have shown that the levels of IL-12 and TNF-*α* are significantly increased in the peripheral blood of patients with IBD [25, 26, 35]. The expression of IL-12 mRNA in the colonic mucosa of patients with UC is significantly higher during the active phase of the disease, and the degree of the increase correlates with the severity of the disease [36, 37]. Genomewide association studies have identified an association between polymorphisms of IL-10 and increased susceptibility to IBD [38–40]. The function of anti-inflammatory macrophages is altered in IL-10R-deficient patients with IBD [41]. IL-10 expression in colonic epithelial cells is elevated in patients with UC [42]. A study showed that IFN-*β* suppressed inflammation induced by high levels of IL-13 in patients with UC [43]. Another study showed that natural IFN-*β* was effective in treating patients with active, steroid refractory UC [44]. In this way, inhibiting the TLR2/NF-*κ*B signaling pathway by modulating the key mediators of this pathway, such as TLR2, IRAK1, IKK-*β*, and NF-*κ*B, and downstream effector cytokines, such as TNF-*α*, IL-12, IL-10, and IFN-*β*, may provide effective treatments for UC.

Herb-partitioned moxibustion is beneficial for patients with UC [45]. This treatment can reduce the expression of IL-8 and ICAM-1 mRNA and proteins in the colonic mucosa of patients with UC [46]. Previous studies have found that IL -12 and TNF-*α* expressions are significantly higher in UC rat colons, but herb-partitioned moxibustion reduces their expression [47]. Herb-partitioned moxibustion can also reduce the concentrations of IL-1*β*, IL-6, and TNF-*α* from induced peripheral blood mononuclear cells (PBMCs) cultured supernatants of UC rats, relieving intestinal inflammation [48]. In the present study, results showed that, in UC rat colons, the normal mucosa structure was damaged and the levels of TLR2, IRAK1, and IKK-*β* mRNA and proteins were significantly higher, and the levels of IL-10 and IFN-*β* proteins were significantly lower than those of the control rat colon. With herb-partitioned moxibustion treatment, the levels of TLR2, IRAK1, and IKK-*β* mRNA and proteins were significantly lower, and the levels of IL-10 and IFN-*β* proteins were significantly higher. These data suggest that the TLR2/NF-*κ*B signaling pathway is involved in the development of UC, and herb-partitioned moxibustion can reverse the dysregulated expression of signaling molecules in the TLR2 pathway effectively, thereby inhibiting TLR2/NF-*κ*B pathway activation, modulating the local immune response in the colonic mucosa, protecting the mucosa, and promoting repair of injured mucosa.

## Figures and Tables

**Table 1:** The gene sequences of the primers.

ID	Primer name	Sequence (5′ to 3′)	Base number
gi|392350511|ref|XM_003750630.1|	TLR2-domain	CCCAAGCACACTCACTCAACT	20
gi|189011593|ref|NM_001127555.1|	IRAK-1	CAAGGAGGCACTACCAGAGAAT	22
gi|158508715|ref|NM_053355.2|	IKK-*β*	CCAAGAGACCAAAGGACAGAAG	22
